# Activating PPARβ/δ Protects against Endoplasmic Reticulum Stress-Induced Astrocytic Apoptosis via UCP2-Dependent Mitophagy in Depressive Model

**DOI:** 10.3390/ijms231810822

**Published:** 2022-09-16

**Authors:** Juan Ji, Shangze Li, Zikai Jiang, Jianbing Yu, Yuqin Sun, Zhenyu Cai, Yinfeng Dong, Xiulan Sun

**Affiliations:** 1 Department of Pharmacology, Neuroprotective Drug Discovery Key Laboratory, Jiangsu Key Laboratory of Neurodegeneration, Center for Global Health, Nanjing Medical University, Nanjing 211166, China; 2 Nanjing University of Chinese Medicine, The Affiliated Hospital of Nanjing University of Chinese Medicine, Nanjing 210023, China

**Keywords:** peroxisome proliferator-activated receptor β/δ, astrocyte, oxidative stress, mitophagy, endoplasmic reticulum stress, depression

## Abstract

As energy metabolism regulation factor, peroxisome proliferator-activated receptor (PPAR) is thought to be a potential target for the treatment of depression. The present study was performed to evaluate the effects of activating PPARβ/δ, the most highly expressed subtype in the brain, in depressive in vivo and in vitro models. We observed that PPARβ/δ agonist GW0742 significantly alleviated depressive behaviors in mice and promoted the formation of autophagosomes around the damaged mitochondria in hippocampal astrocytes. Our in vitro experiments showed that GW0742 could reduce mitochondrial oxidative stress, and thereby attenuate endoplasmic reticulum (ER) stress-mediated apoptosis pathway via inhibiting IRE1α phosphorylation, subsequently protect against astrocytic apoptosis and loss. Furthermore, we found that PPARβ/δ agonist induces astrocytic mitophagy companied with the upregulated UCP2 expressions. Knocking down UCP2 in astrocytes could block the anti-apoptosis and pro-mitophagy effects of GW0742. In conclusion, our findings reveal PPARβ/δ activation protects against ER stress-induced astrocytic apoptosis via enhancing UCP2-mediated mitophagy, which contribute to the anti-depressive action. The present study provides a new insight for depression therapy.

## 1. Introduction

Depression is a common mental disorder characterized by depressed mood, anhedonia, unresponsiveness, decreased volitional activity and cognitive dysfunction. Approximately 300 million people worldwide suffer from depression, and more than 800,000 people commit suicide due to depression every year [1]. The selective serotonin reuptake inhibitors (SSRIs) have been widely used in clinical treatment of depression for more than 40 years; however, due to the slow onset, poor efficacy, and fail to treat patients with major depression [2], it is urgent to understand the etiology and pathophysiology of depression and to seek a new effective target for treatment.

Astrocytes are the most abundant cell types in the central nervous system (CNS), and involved in regulating neurotransmitter homeostasis, synaptic plasticity, energy metabolism and neurotrophic support [3]. Astrocytes play a critical role in antioxidant defense in brain [3,4,5]. However, when the levels of reactive oxygen species (ROS) exceed astrocytic antioxidant capacity, pathological oxidative stress would induce endoplasmic reticulum stress and thereby induce cellular apoptosis. Importantly, reduced astrocyte density has been reported in the brains of depressed individuals and animal models [3,6,7,8], accompanied by reduced in expression of glutamate transporters and release of neurotrophic factors. Therefore, these evidence suggest that astrocytic loss and dysfunction in depression may be related to oxidative stress response.

Endoplasmic reticulum (ER) is a critical organelle in charge of protein synthesis, transport and folding, calcium regulation, lipid and sterol synthesis, which are crucial for cellular homeostasis [9]. Alterations in the ER folding environment cause the accumulation of misfolded proteins in the ER lumen. It leads to ER stress, which restores ER homeostasis through activating unfolded protein response (UPR) [10,11,12]. However, when ER stress is chronic or too severe, it can engage apoptosis. Current research has found that the levels of ER stress proteins GRP78, GRP94 and calreticulin are elevated in the postmortem temporal cortex of patients with depression [13,14]. Similarly, Nevell et al. [15] demonstrated that systemic and persistent activation of ER stress is associated with depression. Recently, reactive oxygen species (ROS) have emerged as crucial regulators of ER function and UPR activation in several diseases. And in those diseases, ER stress and increased ROS production occur concurrently [9,16,17,18,19]. However, the molecular mechanism between oxidative stress and ER stress is still obscure. Moreover, how ROS modulates the ER-mitochondria crosstalk during ER stress-induced apoptosis has not been fully investigated [9,10,16,17], especially in depression.

Peroxisome proliferator-activated receptors (PPARs) are members of the nuclear receptor superfamily, including three subtypes, PPARα, PPARβ/δ and PPARγ [20]. Its physiological functions are related to metabolism, energy homeostasis, cell development and differentiation. PPARs agonists have been used for the treatment of metabolic diseases, especially dyslipidemia and type 2 diabetes mellitus (T2DM) [21]. Notably, many studies found that activation of PPARγ improves mood disorders, especially in major depressive disorder and bipolar disorder [22,23,24,25]. PPARγ agonists such as rosiglitazone and pioglitazone showed an antidepressant-like activity [26,27,28,29,30]. The expression of PPARβ/δ in the brain is higher than the other two subtypes. A recent study has shown that PPARβ/δ expression in the hippocampus was markedly decreased in the depressive model mice [31,32]. Activation of PPARβ/δ ameliorated depression-like behaviors in mice [33]. However, the detailed impacts of PPARβ/δ in the depression remain unclear. Therefore, the aim of the present study was to demonstrate the mechanisms involved in the anti-depressive effects of PPARβ/δ activation in the unpredictable chronic mild stress (UCMS)-induced depressive mouse model and corticosterone-induced depressive cellular model.

## 2. Results

### 2.1. Activating PPARβ/δ Ameliorates Depression-Like Behavior in Mice

**Figure 1:**
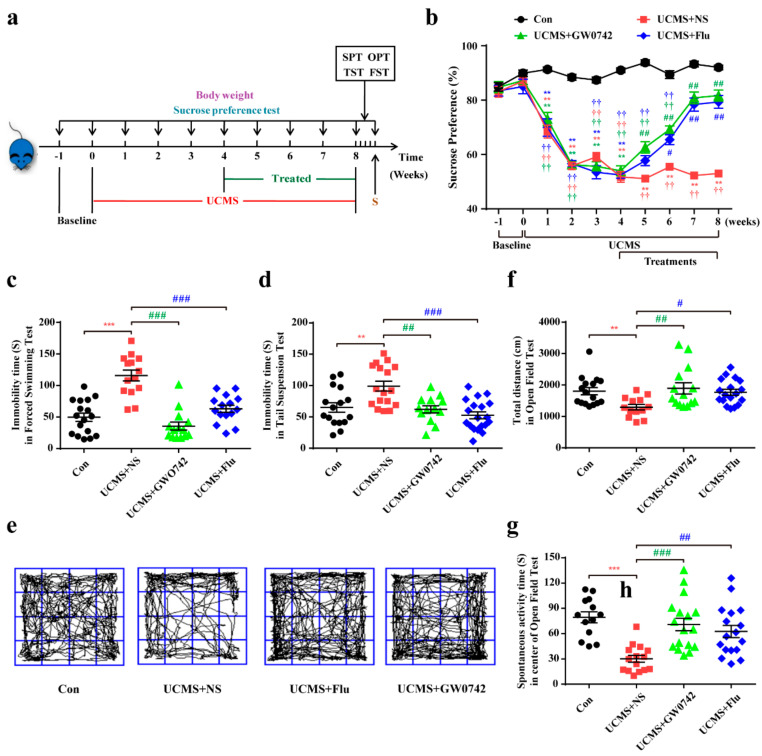
GW0742 ameliorates depression-like behavior in mice. (**a**) Experiment schedule for in vivo experiments. After 4 weeks of UCMS exposure, mice were divided into three groups and respectively injected with saline (10 mg/kg), GW0742 (10 mg/kg) and fluoxetine (10 mg/kg). Subsequently, behavioral tests were performed after 4 weeks of drugs treatment. (**b**) The continuous changes of SPT are detected during the experiment. Data is presented as means ± SEM. n ≥ 20 per group. ** *p* < 0.01, compared to Con group at each time point; ^#^ *p* < 0.05, ^##^ *p* < 0.01, compared to UCMS group at each time point; ^††^ *p* < 0.01 compared to the base line in each group, by two-way ANOVA and Tukey’s test. (**c**,**d**) The immobility time of mouse is shown in FST and TST. (**e**) The motion curve of mouse is shown in OFT. (**f**) The total distance and (**g**) the spontaneous activity time in the center area is measured in OFT. Data is presented as means ± SEM. n ≥ 13 per group. ** *p* < 0.01, *** *p* < 0.001, compared to Con group; ^#^ *p* < 0.05, ^##^ *p* < 0.01, ^###^ *p* < 0.001, compared to UCMS group, by one-way ANOVA and Tukey’s test.

Compared with the control group, UCMS-induced depressive mice show less preference in sucrose intake whereas GW0742 or fluoxetine treatment reversed the reduction in sucrose consumption induced by UCMS (Figure 1b). FST, TST and OFT are often performed to evaluate the depression-like behaviors, indicating behavioral despair and social phobia, respectively. After the administration of GW0742 or fluoxetine for 4 weeks in the UCMS group, the duration of immobility during FST (Figure 1c) and TST (Figure 1d) remarkably decreased. The total travelled distance and time spent in the central region showed a significant increase in GW0742 or fluoxetine-treated groups, suggesting that those mice were more willing to explore the environment than the depressive model mice (Figure 1e–g).

### 2.2. Activating PPARβ/δ Ameliorates Astrocytic Injury in In Vivo and In Vitro Depressive Models

**Figure 2:**
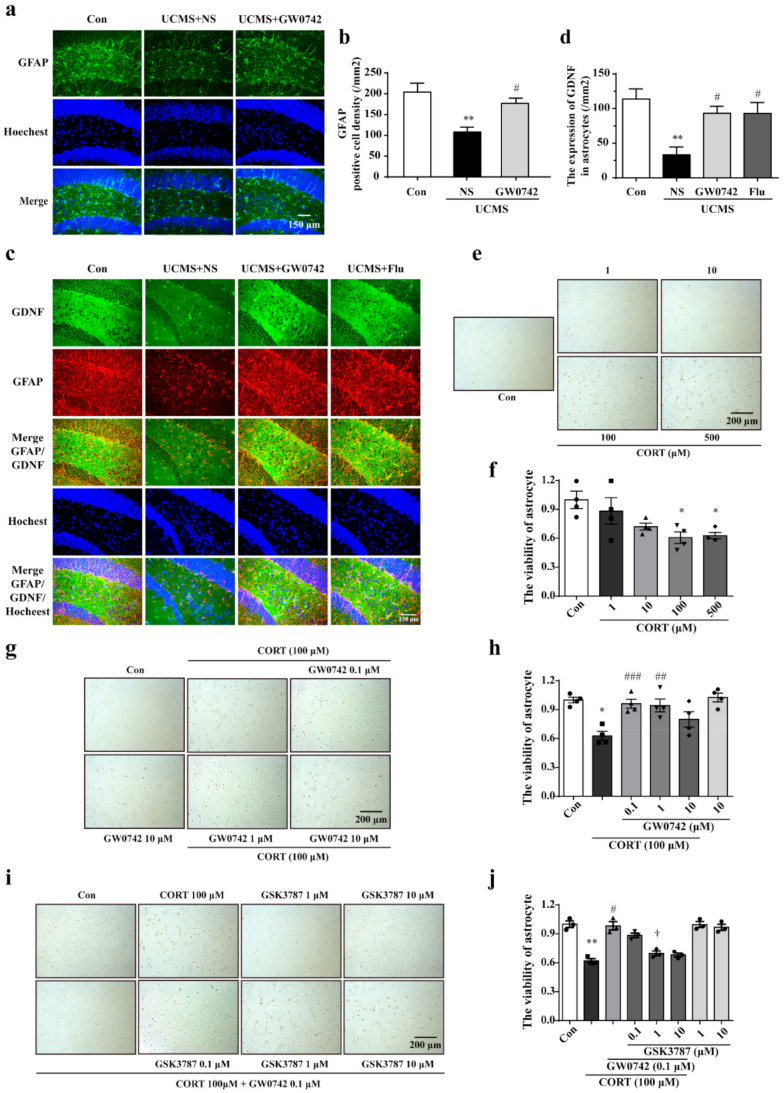
GW0742 a ameliorates astrocytic injury in in vivo and in vitro *depressive models*. (**a**) GFAP is used to label astrocyte in hippocampus region. PPARβ/δ agonist reduces the loss of astrocytes in hippocampus of UCMS treated mice. (**b**) Quantitative analysis of GFAP^+^. Data is presented as means ± SEM. n = 5, ** *p* < 0.01 vs. Con group; ^#^ *p* < 0.05 vs. UCMS group, by one-way ANOVA and Tukey’s test. (**c**) GDNF and GFAP are co-located in the hippocampus region. GW0742 promotes astrocytes to secrete GDNF in hippocampus of UCMS treated mice. (**d**) Quantitative analysis of GDNF expression in astrocytes. Data is presented as means ± SEM. n = 5, ** *p* < 0.01 vs. Con group; ^#^ *p* < 0.05 vs. CMS group, by one-way ANOVA and Tukey’s test. (**e**,**f**) The astrocytes are treated with CORT at the concentrations of 1, 10, 100 and 500 μM for 24 h. Data is presented as means ± SEM. n = 5, * *p* < 0.05 vs. Con group, by T test. (**g**,**h**) The astrocytes are treated with CORT (100 μM) and GW0742 (0.1, 1, and 10 μM). Data is presented as means ± SEM. n = 5, * *p* < 0.05 vs. Con group; ^##^ *p* < 0.01, ^###^ *p* < 0.001 vs. CORT group, by one-way ANOVA and Tukey’s test. (**i**,**j**) The astrocytes are treated with CORT (100 μM), GW0742 (0.1 μM) and GSK3787 (0.1, 1, 10 μM). Data is presented as means ± SEM. n = 4, ** *p* < 0.01 vs. Con group; ^#^ *p* < 0.01 vs. CORT group; ^†^ *p* < 0.05 vs. CORT + GW0742 group, by one-way ANOVA and Tukey’s test.

Using glial fibrillary acidic protein (GFAP) to label astrocyte, we observed a large loss of astrocytes in the hippocampus of UCMS treated mice, which was consistent with literature reports. PPARβ/δ agonist GW0742 significantly reversed the decreases in astrocytes (Figure 2a,b). Simultaneously, treatment with GW0742 significantly increased the co-localization of GFAP and glia-derived neurotrophic factor (GDNF) (yellow staining) (Figure 2c,d), suggesting that PPARβ/δ activation also improved the protection for astrocytes. In the primary cultured astrocytes, 100 µM of corticosterone significantly decreased the cellular viability (Figure 2e,f). Consistently, the viability of astrocytes was restored by treatment with 0.1 µM PARβ/δ agonist GW0742 (Figure 2g,h). The protection of GW0742 were prevented by the PARβ/δ antagonist GSK3787 at a concentration of 1 µM (Figure 2i,j). The findings confirm that PARβ/δ agonist protects astrocytes against the injury in depressive models.

### 2.3. Activating PPARβ/δ Ameliorates Oxidative Stress-Induced Damages in Astrocytes

**Figure 3:**
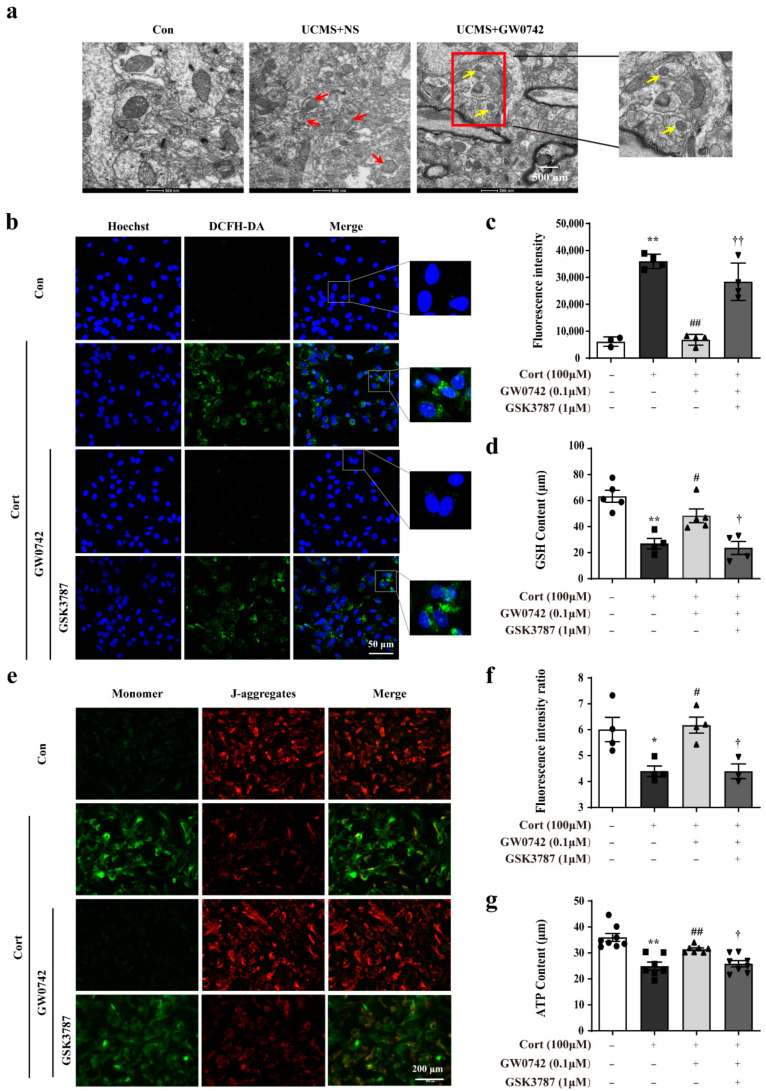
GW0742 ameliorates oxidative stress-induced damages in astrocytes. Representative TEM images are used to observe the structure of mitochondria (**a**) in hippocampal astrocytes. Scale bar = 2 µm and 500 nm. The structure of damaged mitochondria is shown as the red arrows. The formation of autophagosomes is shown as yellow arrows. After staining astrocytes with DCFH-DA probe, the fluorescence intensity of ROS was captured under fluorescence microscope (**b**) and the Fluorescence intensity of ROS was quantified by microplate system (**c**). Scale bar = 200 µm. Data is presented as means ± SEM. n = 6, ** *p* < 0.01 vs. Con group, ^##^ *p* < 0.01 vs. CORT group, ^††^ *p* < 0.01 vs. CORT + GW0742 group, by one-way ANOVA and Tukey’s test. (**d**) GSH levels were evaluated by GSH assay kit. Data is presented as means ± SEM. n = 5, ** *p* < 0.01 vs. Con group, ^#^ *p* < 0.05 vs. CORT group, ^†^ *p* < 0.05 vs. CORT + GW0742 group, by one-way ANOVA and Tukey’s test. (**e**) The colocalization of JC-1 (red) and monomer (green) was reflected the changes in mitochondrial membrane potential. Scale bar = 200 µm. (**f**) Quantitative analysis of JC-1 fluorescence intensity. Data is presented as means ± SEM. n = 5, * *p* < 0.05 vs. Con group, ^#^ *p* < 0.05 vs. CORT group, ^†^ *p* < 0.05 vs. CORT + GW0742 group, by one-way ANOVA and Tukey’s test. (**g**) ATP content was determined by means of the luciferase assay system using an ATP assay kit. Data is presented as means ± SEM. n = 5, ** *p* < 0.01 vs. Con group, ^##^ *p* < 0.01 vs. CORT group, ^†^ *p* < 0.05 vs. CORT + GW0742 group, by one-way ANOVA and Tukey’s test.

In the mice exposed to UCMS, a large number of damaged mitochondria were observed via transmission electron microscopy, accompanied by mitochondrial swelling, disordered arrangement of cristae, and mitochondrial fusion (showing as red arrows, Figure 3a). Treatment with GW0742 could significantly ameliorate the UCMS-induced structural damages of mitochondria in hippocampal astrocytes (Figure 3a).

### 2.4. Activating PPARβ/δ Enhances Mitophagy in Astrocytes via Upregulating the Expression of UCP2

**Figure 4:**
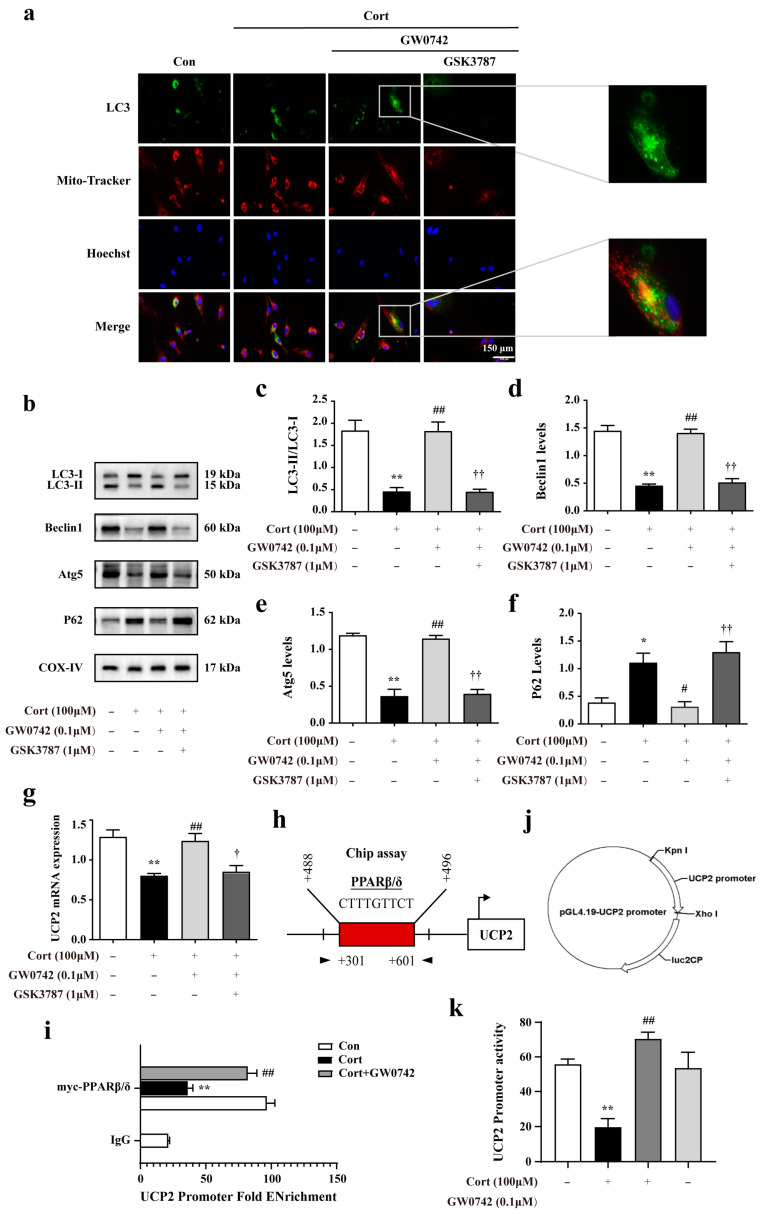
GW0742 enhances mitophagy in astrocytes via upregulating the expression of UCP2. (**a**) Fluorescence microscope analysis was used to measure autophagosomes in mitochondria by monitoring the colocalization of LC3 and Mito-Tracker. Scale bar = 150 µm. (**b**–**f**) Expression levels of proteins regulating mitophagy in astrocytes following diverse stimulation. Data is presented as means ± SEM. n ≥ 4, * *p* < 0.05, ** *p* < 0.01 vs. Con group, ^#^ *p* < 0.05, ^##^ *p* < 0.01 vs. CORT group, ^††^ *p* < 0.01 vs. CORT + GW0742 group, by one-way ANOVA and Tukey’s test. (**g**) UCP2 mRNA expressional changes were detected in astrocytes after various stimulation. Data is presented as means ± SEM. n ≥ 4, ** *p* < 0.01 vs. Con group, ^##^ *p* < 0.01 vs. CORT group, ^†^ *p* < 0.05 vs. CORT + GW0742 group, by one-way ANOVA and Tukey’s test. (**h**) The site shows PPARβ/δ binds to UCP2 promoter region. (**i**) ChIP-qPCR assay was used to detect the binding levels of PPARβ/δ at the UCP2 promoter regions. n ≥ 3, ** *p* < 0.01 vs. Con group; ^##^ *p* < 0.01 vs. Cort group; by one-way ANOVA and Tukey’s test. (**j**) The plasmid of UCP2 promoter combined with Renilla/firefly luciferase constructs. (**k**) The luciferase reporter was used to detect the activity of UCP2 promoter. The data presented are the relative expression level of firefly luciferase standardized to Renilla luciferase. n ≥ 4, ** *p* < 0.01 vs. Con group; ^##^ *p* < 0.01 vs. Cort group; by one-way ANOVA and Tukey’s test.

In order to explore the mechanism involved in the anti-oxidative effects of PPARβ/δ activation in the astrocytes, we further found that GW0742 significantly enhanced autophagosome formation and phagocytosing the damaged mitochondria. As shown in Figure 4a, a larger number of colocalization sites for LC3 and Mito-Tracker were observed in the GW0742-treated group compared to corticosterone-treated group. In addition, the reductions in LC3-II/LC3-I, Beclin1 and Atg5 expressions and the increases in p62 protein were restrained by PPARβ/δ agonist GW0742 (Figure 4b–f). These findings reveal that PARβ/δ activation can clear up the damaged mitochondria by promoting mitophagy.

Uncoupling protein 2 (UCP2) is a mitochondrial inner-membrane protein, which plays a critical role in mitochondrial function, including control of reactive oxygen species (ROS) generation and fuel utilization. UCP2 is a canonical target gene of PPARβ/δ [34]. We found that PPARβ/δ activation dramatically reversed the decreasing of UCP2 mRNA levels by corticosterone (Figure 4g). A chromatin immunoprecipitation (ChIP) assay performed in the astrocytes infected with a myc-tagged PPARβ/δ DNA construct showed that PPARβ/δ bound to the UCP2 promoter (Figure 4h,i). The results from double luciferase reporter gene test revealed that GW0742 significantly enhanced the lower UCP2 promoter activity (Figure 4k). In summary, we find that PPARβ/δ activation promotes mitophagy by binding to UCP2 promoter, which increases the activity of UCP2 promoter and the expression of UCP2.

### 2.5. Activating PPARβ/δ Ameliorates Mitochondrial Damage via UCP2-Mediated Mitophagy in Astrocytes

**Figure 5:**
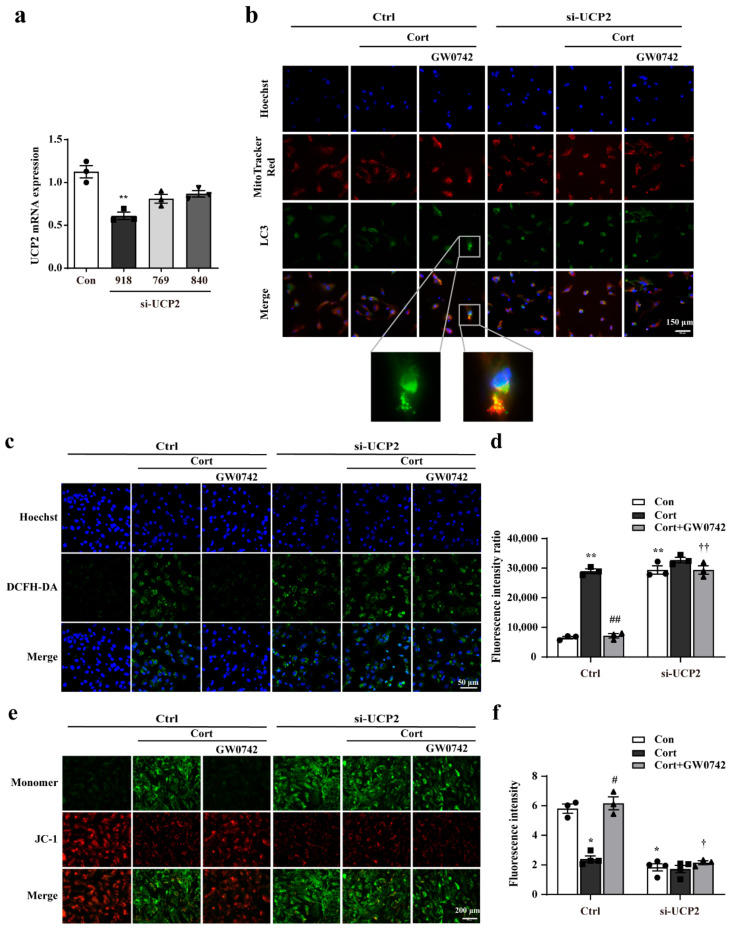
GW0742 ameliorates mitochondrial damage via UCP2-mediated mitophagy in astrocytes. (**a**) mRNA levels of UCP2 were detected by RT-qPCR after UCP2-specific siRNA transfection in astrocytes. Data is presented as means ± SEM. n ≥ 4, ** *p* < 0.01 vs. Con group, by T test. (**b**) After treatment with UCP2 siRNA, the colocalization of LC3 and Mito-Tracker was measured in astrocytes. Scale bar = 150 µm. (**c**) The fluorescence intensity of ROS was quantified by microplate system and (**d**) the Fluorescence intensity of ROS was captured under fluorescence microscope. Scale bar = 150 µm. Data is presented as means ± SEM. n ≥ 5, ** *p* < 0.01 vs. Con group in the Ctrl; ^##^ *p* < 0.01 vs. CORT group in the Ctrl; ^††^ *p* < 0.01 vs. CORT + GW0742 group in the Ctrl, by two-way ANOVA and Tukey’s test. (**e**) The colocalization of JC-1 (red) and monomer (green) was reflected the changes in mitochondrial membrane potential. Scale bar = 200 µm. (**f**) Quantitative analysis of JC-1 fluorescence intensity. Data is presented as means ± SEM. n ≥ 4, * *p* < 0.05 vs. Con group in the Ctrl; ^#^ *p* < 0.05 vs. CORT group in the Ctrl; ^†^ *p* < 0.05 vs. CORT + GW0742 group in the Ctrl, by two-way ANOVA and Tukey’s test.

Furtherly, we used siRNA to knockdown UCP2 to confirm that UCP2 is a key regulatory protein of PPARβ/δ activation-mediated protective effects. Compared to siRNA-UCP2 (769) and siRNA-UCP2 (840), transfection with siRNA-UCP2 (918) resulted in a 50% suppression of UCP2 expression (Figure 5a). Then, UCP2 siRNA (918) was used for transfection in the subsequent experiments. Figure 5b showed that knocking down UCP2 prevented mitophagy increased by GW0742 in the astrocytes.

Coincidentally, knocking down UCP2 reversed the decrease in ROS and the increase in JC-1 fluorescence improved by PPARβ/δ activation (Figure 5c,e). Microplate system analysis also showed knocking down UCP2 reversed the GW0742-induced changes of ROS and mitochondrial membrane potential (MPP) (Figure 5d,f).

### 2.6. Activating PPARβ/δ Reduces ER Stress-Induced Astrocytic Apoptosis Induced by Corticosterone

**Figure 6:**
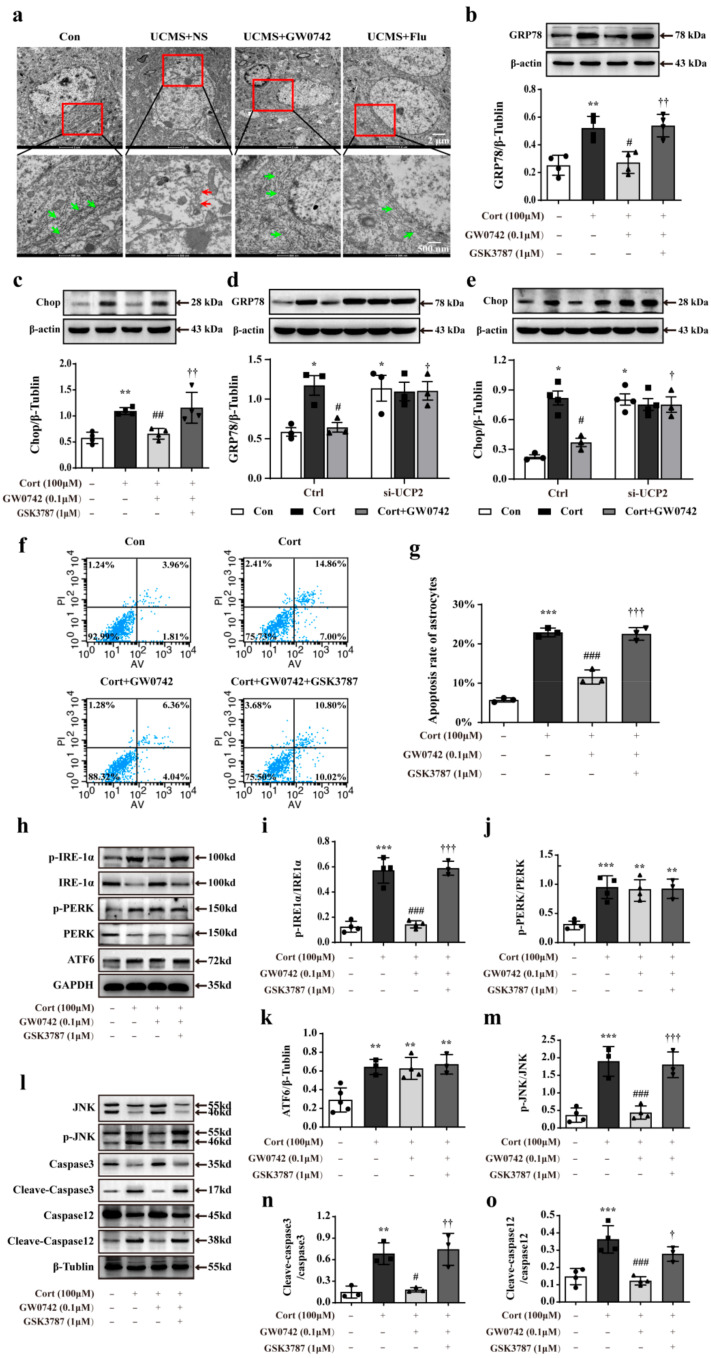
GW0742 reduces ER stress-induced astrocytic apoptosis induced by corticosterone. Representative TEM images are used to observe the structure of ER (**a**) in hippocampal astrocytes. Scale bar = 2 µm and 500 nm. The structure of the normal ER is shown as the green arrows. The structure of damaged ER is shown as the red arrows. GRP78 (**b**) and Chop (**c**) expressions are measured by western blot. Data is presented as means ± SEM. n ≥ 4, ** *p* < 0.01 vs. Con group; ^#^ *p* < 0.05; ^##^ *p* < 0.01 vs. CORT group; ^††^ *p* < 0.01 vs. CORT + GW0742 group, by one-way ANOVA and Tukey’s test. (**d**,**e**) Knocking down UCP2 turned over the protein expressions of GRP78 and Chop in astrocytes treated with corticosterone and GW0742. Data is presented as means ± SEM. n ≥ 3, * *p* < 0.05 vs. Con group in the Ctrl; ^#^ *p* < 0.05 vs. CORT group in the Ctrl; ^†^ *p* < 0.05 vs. CORT + GW0742 group in the Ctrl, by two-way ANOVA and Tukey’s test. (**f**,**g**) Quantitative analysis of the apoptosis rate in astrocytes. Data is presented as means ± SEM. n = 5, *** *p* < 0.001 vs. Con group; ^###^ *p* < 0.001 vs. CORT group; ^†††^ *p* < 0.001 vs. CORT + GW0742 group, by one-way ANOVA and Tukey’s test. (**h**–**o**) Western blotting is used to analyze the expression of p-IRE1α/IRE1α, p-PERK/PERK, AFT6, p-JUK/JUK, C-Caspase3/Caspase3 and C-Caspase12/Caspase12 among the different group. Data is presented as means ± SEM. n ≥ 4, ** *p* < 0.01, *** *p* < 0.001 vs. Con group; ^##^ *p* < 0.01 vs. CORT group; ^†^ *p* < 0.05, ^††^ *p* < 0.01 vs. CORT + GW0742 group, by one-way ANOVA and Tukey’s test.

Excessive ROS and the accumulation of damaged mitochondria would trigger excessive and prolonged ER stress, and thereby cellular apoptosis. In the mice exposed to UCMS, the structures of ER in hippocampal astrocytes were severely damaged, showing fragmentation of membrane and reticular cavity, as well as loss of ribosomes (showing as red arrows) observed via transmission electron microscopy (Figure 6a). In the primary cultured astrocytes, PPARβ/δ activation significantly prevented the corticosterone-induced increases in the expressions of glucose regulated protein 78 (GRP78) and CCAAT/enhancer-blinging protein homologous protein (CHOP), which were reversed by the PPARβ/δ antagonist GSK3787 (Figure 6b,c). Knocking down UCP2 significantly inhibited the downregulation of GRP78 and CHOP in GW0742-treated astrocytes (Figure 6d,e). These results strongly suggest that PPARβ/δ agonist reduces the ER stress in the astrocytes.

As shown in Figure 6f,g, corticosterone caused astrocytic apoptosis, which were suppressed by treatment with GW0742. In mammals, there are three transmembrane proteins for ER stress-induced apoptosis pathway, namely RNA-dependent protein kinase-like ER eukaryotic initiation kinase (PERK), inositol-requiring ER-to-nucleus signaling protein 1α (IRE1α) and activating transcription factor 6 (ATF6). We found that PPARβ/δ agonist only inhibited IRE1α phosphorylation against corticosterone-mediated astrocytic apoptosis and failed to affect the phosphorylation of PERK and ATF6 (Figure 6h–k). Additionally, we detected the related proteins in the signaling pathway of IRE1α activation-mediated apoptosis. The results showed that PPARβ/δ activation could potentially inhibit the phosphorylation of JNK and reduce the levels of cleaved-caspase12 and cleaved-caspase 3 (Figure 6l–o). Therefore, our results reveal that PPARβ/δ agonist can prevent ER stress-induced astrocytic apoptosis via inhibiting IRE1α-pJNK-caspase 12-caspase 3 signal pathway.

## 3. Discussion

Many studies have demonstrated that PPARγ is an important therapeutic target for depression [27,35]. Our previous studies also have revealed rosiglitazone exerted anti-depressive effects by maintaining basic neuronal autophagy and inhibiting excessive astrocyte apoptosis [26]. Now, many PPARγ agonists to address major depressive disorder, have tested in a few clinical trials. Based on the fact that PPARβ/δ is the most richly expressed subtype in the brain, it will be more meaningful to explore its regulatory role in depression. Li et.al provided promising and novel evidence that hippocampal PPARδ is an important therapeutic target for depression [31]. They found up-regulating hippocampal PPARδ by telmisartan results in an anti-depressive effect through the elevation of 5-HTT expressions. Similarly, our study showed that PPARβ/δ agonist GW0742 effectively raised sucrose intake in the SPT, and locomotor activity and frequency in the OFT, as well as decreased immobility time in FST and TST. The present work confirmed that PPARβ/δ activation could ameliorate the symptoms of depression, and the therapeutic effects of GW0742 were similar to fluoxetine used in clinic for depression.

In the brain, astrocytes are the most numerous cell types and perform many functions that are essential for the normal activity of neurons. It has been reported that postmortem brain samples from patients with depression showed a decline number of astrocytes in the prefrontal cortex and the hippocampus [36]. Selectively acute injury to astrocytes is adequate to induce depressive-like behaviors. Thus, protecting astrocytes to maintain neuronal survival and function may be a potential treatment strategy for depression. However, whether activating PPARβ/δ could protect the damaged astrocytes in depression is still unclear. Our results showed that GW0742 increased the number of GFAP-labeled astrocytes and the levels of GDNF in the hippocampus of UCMS-treated mice. Consistently, GW0742 prevented against corticosterone-induced vitality decline in astrocytes, which was blocked by PPARβ/δ antagonist GSK3787. Therefore, our results suggest that PPARβ/δ agonist protects against astrocytic injury in the depression.

Accumulating evidence has demonstrated that the pathogenesis of depression is associated with the oxidative stress [37,38,39,40]. In physiological or pathological conditions, mitochondrial metabolism is critical for the regulation of redox in astrocytes. The endoplasmic reticulum (ER) as an important platform for initiating, receiving, and transmitting redox signals is also a significant source of ROS [37,41]. Excessive ROS can lead to mitochondrial dysfunction and endoplasmic reticulum stress. Subsequently, the accumulation of damaged mitochondria and ER further increases the levels of ROS, creating a vicious cycle [42,43,44]. In the present study, we found the structure of mitochondria and ER were significantly impaired in hippocampal astrocytes of UCMS-treated mice. GW0742 treatment could reduce the damages and maintain the normal structure of mitochondria and ER in hippocampal astrocytes. Consistently, treatment with GW0742 effectively attenuated corticosterone-induced increases in GRP78, CHOP, ROS and GSH, and reductions in mitochondrial membrane potential and ATP level in astrocytes. The above effects of GW0742 were blocked by PPARβ/δ antagonist GSK3787. Collectively, both in vivo and in vitro, our data reveals that activating PPARβ/δ inhibits intracellular ROS production and exerts a protective effect against mitochondrial damage and ER stress in astrocytes. It may provide new insights for depression therapy. As is known to all, ROS signaling pathways are shown to facilitate downstream cell death and organ dysfunction in the face of chronic ER stress, leading to disease states [9,10,16,17]. RNA-dependent protein kinase-like ER eukaryotic initiation kinase (PERK), inositol-requiring ER-to-nucleus signaling protein 1α (IRE1α) and activating transcription factor 6 (ATF6) are the three important transmembrane proteins that trigger ER stress-induced apoptosis [9,16,18]. More specifically, we found that PPARβ/δ activation prevented ER stress-mediated apoptosis pathway by inhibiting the phosphorylation of IRE1α, thereby plays a protective role in astrocytes.

Autophagy, including mitophagy, is a tightly modulated cellular degradation pathway by which defective proteins, impaired organelles, and other cellular constituents are sequestered in autophagosomes and delivered to lysosomes for degradation. Moderate autophagy is generally considered as a self-protection mechanism against cellular damage caused by intracellular and extracellular stress. Brittany et al. [45] pointed out that autophagy is not only crucial in regulating cellular and mitochondrial oxidative stress, but also able to attenuate ER-stress by degrading aggregated/misfolded proteins. Their data nicely demonstrates that autophagy in protecting myoblasts from mitochondrial oxidative stress and apoptotic signaling during differentiation [45]. Moreover, there is increasing evidence that mitophagy is important for the homeostasis and function of mitochondria and ER [19,43,45]. Notably, the results of electron microscope showed that the increased autophagosomes appeared in hippocampal astrocytes treated by GW0742, surrounded by the damaged mitochondria. Moreover, our in vitro results showed that GW0742 induced the significant colocalization of LC3 puncta and Mito-tracker in astrocytes, increased LC3-II/LC3-I, Beclin1, and Atg5, but decreased p62 expressions. Therefore, our data suggest that PPARβ/δ activation-mediated mitophagy contributes to the alleviation of mitochondrial damage and ER stress.

It has been reported that UCP2 could be upregulated by stress, injury or ischemia, and overexpression of UCP2 plays neuroprotective effects against oxidative stress in vivo and in vitro [34,46,47]. As the canonical target gene of PPARβ/δ [48], whether PPARβ/δ activation could promote astrocytic mitophagy via targeting UCP2 is not understood totally. For the first time, we found GW0742 enhanced the UCP2 promoter activity and increased the expressions of UCP2 in astrocytes. Moreover, UCP2 knockdown could significantly inhibit GW0742-induced the reduction in GRP78 expression, CHOP expression, and ROS content, as well as increase in mitochondrial membrane potential. UCP2 knockdown also blocked the GW0742-mediated pro-mitophagy effects. These results demonstrate that up-regulation of UCP2 expression is necessary for PPARβ/δ activation -mediated mitophagy.

**Figure 7:**
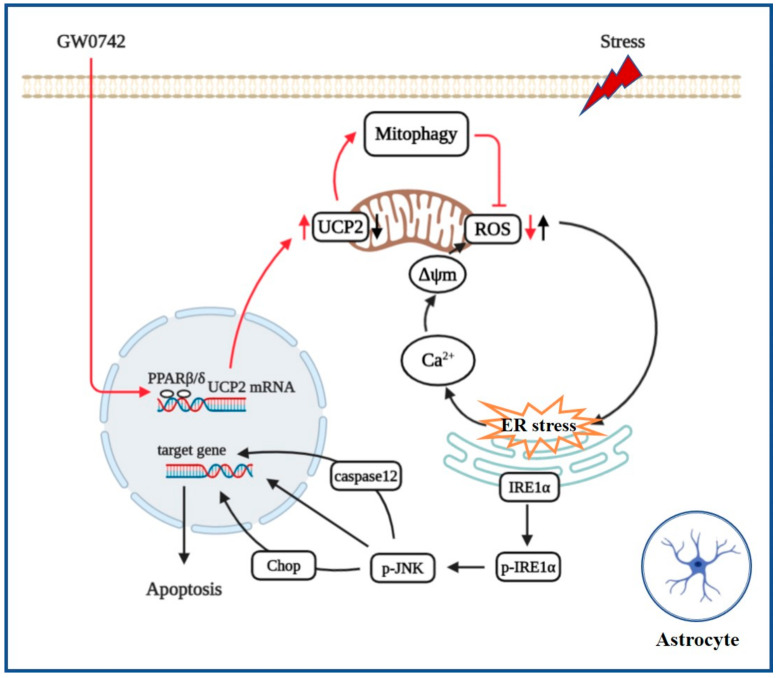
PPARβ/δ agonist protects against oxidative stress and ER stress-induced astrocytic apoptosis by inhibiting the phosphorylation of IRE1α, which are due to enhancing UCP2-dependent mitophagy.

Taken together, this study reveals that PPARβ/δ agonist targets UCP2-mediated mitochondrial autophagy against the cascade of oxidative stress and ER stress induced astrocytic damage (Figure 7). These results provide a promising molecular target for the development of antidepressant drugs besides regulating neurotransmitters.

## 4. Materials and Methods

### 4.1. Experimental Animals and Procedures

Eight weeks old C57B/6J male mice were obtained from Beijing Vital River Laboratories Animal Technology Company. All of the animal operational procedures were performed in accordance with the Institution for Animal Care and Use Committee and approved by Animal Core Facility of Nanjing Medical University (approval No. NJMU-1807003). All mice adapted to the new environment for one week before the onset of the experiments. Then, they were randomly divided into 4 groups: (i) Control (no stress); (ii) UCMS (stress + normal saline); (iii) UCMS + GW0742 (stress + PPARβ/δ agonist); (iv) UCMS + fluoxetine (stress + fluoxetine), according to their body weight and sugar water preference rate. Unpredictable chronic mild stress is known to be an effective method for inducing depression-like behavior. To create a UCMS model, we exposed the experimental mice to a number of stressors, including rhythm disturbance, strong light, flashing lights and noise stimulation, wet cage, 45° inclined cage, crowded, empty cage, clip tail, tail water, bound water, shaking table. After 4 weeks of CMS modeling, the treatment group was treated with daily intraperitoneal injection of PPARβ/δ agonist GW0742 (10 mg/kg) and the positive drug fluoxetine (10 mg/kg), respectively. At the same time, the mice in the non-drug treatment group were given intraperitoneal injection of normal saline (NS, 10 mg/kg) for 4 weeks. The detailed experimental protocol is shown in Figure 1a. Finally, animals were euthanized with a high dose of pentobarbital (100 mg/kg), and vital signs were reviewed to confirm death after 10 min. All possible methods were applied to minimize the number of animals and their suffering, in accordance with the principles of 3Rs.

### 4.2. Sucrose Preference Test

Before testing, all groups of mice were deprived of water overnight. Then, two drinking bottles, one of which contains 1% sucrose water and the other contains sterile water, were weighted and placed in the drinking hole of each cage. To avoid the impact of drinking site preference, the sites of the drinking bottles were swapped every 3 h. Twelve hours later, the two bottles were weighed. The weight difference of each bottle reflected the sucrose water or sterile water intake. The SPT value was then calculated using the following formula: SPT (%) = sucrose water intake/(sucrose water intake + sterile water intake) × 100%.

### 4.3. Forced Swimming Test

The forced swimming test (FST) was performed according to the method previously described [49,50] and was employed to evaluate depression-like behavior. After 8 weeks of treatment, each mouse was individually placed into a transparent glass cylinder (45 cm height × 35 cm diameter) containing 40 cm of water at 25 °C. A video camera recorded the movement of mice during a 6-min session, of which the data was analyzed by the Forced Swim Scan (Clever Sys Inc., Reston, VA, USA).

### 4.4. Tail Suspension Test

The tail suspension test (TST) was commonly used to evaluate depression-like behavior, as described previously [49,51]. Briefly, mice were suspended 50 cm above the floor by the tail using tape attached to a hook. The immobility time defined as the period during which each mouse was not struggling. A video camera recorded the motor process of mice during a 6-min session, the immobility time of which was analyzed by the Tail Suspension Scan (Clever Sys Inc., Reston, VA, USA).

### 4.5. Open Field Test

The open field test (OFT) is frequently used to evaluate the ability of locomotor activity and spontaneous exploration for mice [49,50]. The experiment was performed in a stainless square block (49.5 cm × 49.5 cm × 48.3 cm). The total duration that mice moved in the center of the block was recorded as an index of anxiety. Mice were placed one by one randomly into the block and allowed to explore the environment freely for 6 min. The trajectory of mice was recorded by video camera and analyzed by the Open Field Scan (Clever Sys Inc., Reston, VA, USA).

### 4.6. Primary Astrocytes Culture

Primary astrocyte cultures were performed as previously described and were isolated from 1–3 days old postnatal Sprague Dawley rats, which were purchased from Animal Core Facility of Nanjing Medical University. In brief, the cleaned cerebral cortices were minced into a fine slurry using a pair of corneal scissors and digested by 0.25% trypsin/EDTA (Gibco, Grand Island, NY, USA) at 37 °C for 20 min. Suspended cells were filtered (40 μm) and centrifuged for 5 min at 1000 rpm and seeded into poly-D-lysine-coated (0.1 mg/mL; Sigma Chemical, St. Louis, MO, USA) 25-cm^2^ culture flasks. The cultures were maintained for 10 days in Dulbecco’s modified Eagle’s medium (DMEM) (Gibco) containing 10% fetal bovine serum (FBS) (Gibco), 1% penicillin-streptomycin (Gibco) at 37 °C in a humidified 5% CO_2_–95% air atmosphere. Media were replaced one day after preparation and subsequently every 2–3 days. Before the experiments, microglial cells were isolated from the mixed primary culture cells, the percentage of the primary astrocytes was confirmed by GFAP staining with over 97% purity.

### 4.7. Cell Viability Assay

The methyl thiazolyl tetrazolium (MTT) assay was used to assess astrocyte viability through formazan formation measurements. Astrocytes were seeded onto 96-well plates at a density of 5 × 10^4^ cells per well. After 24 h of adherence, the cells were successively treated with different drugs, such as corticosterone and/or GW0742 or GSK3787, and incubated in a CO_2_ incubator for 24 h. Then, 20 μL of MTT at a concentration of 5 mg/mL was added to each well with the medium and incubated for 4 h at 37 °C. Finally, the medium was removed from the wells, and 150 μL of DMSO was added to dissolve the formazan crystals. The absorbance of the colored solutions was quantified using a spectrophotometer with an excitation wavelength of 570 nm (iMark Microplate Reader; Bio-Rad, Hercules, CA, USA). Cell activity was calculated as a percentage according to the following formula: percentage cell viability = (absorbance of the experiment sample/absorbance of the control) × 100.

### 4.8. Tissue Processing and Immunohistochemistry

The rats were perfused transcardially with PBS and 4% paraformaldehyde solution. The brain of each mouse was carefully removed and fixed in 4% paraformaldehyde solution for 2 h and dehydrated in 20–30% sucrose. The cryo-tissues were sliced at 16 µm per section; 6 s were collected on a slide, 6 slides were prepared from a single rat brain, 8–10 animals in each group. The 16 µm thickness were blocked in PBS containing 10% goat serum for 1 h at room temperature and incubated a series of primary antibodies overnight. Primary antibodies included anti-GFAP (Millipore, Cat# MAB3402, 1:200); anti-GDNF (Raybiotech, Inc., Cat# 168-10823, 1:200). Then, the alexa Fluor-conjugated secondary antibodies were used to incubate the samples for 1 h at room temperature, including donkey anti rabbit IgG ALEXA flour 488 (1:1000, #a21206, Invitrogen) and donkey anti mouse IgG ALEXA flour 488 (1:1000, #a21202, Invitrogen), and donkey anti mouse IgM ALEXA flour 594 (1:1000, #715-585-020, jacksonslab). After three more washed, stained nuclei were stained with Hoechst 33342 for 20 min. Quantification of immunohistochemistry results was performed blindly by two experimenters as described in reference. Images were captured specify the brain regions (Nikon A1RSi, Tokyo, Japan). The data are expressed as the number of double positive cells per unit area of analyzed zones.

### 4.9. Immunofluorescent Staining

When cells have reached the desired confluency, remove the media from the dish and add prewarmed (37 °C) staining solution containing MitoTracker^®^ probe (thermos, Cat# M22425, a working concentration of 200 nM) incubation for 30 min in a CO_2_ incubator. Washing the cells. After staining, wash the cells in a fresh, pre-warmed buffer or growth medium. The cells were fixed in 4% paraformaldehyde solution at room temperature for 30 min and were blocked in 3% BSA containing 0.1% Triton X-100 for 1 h at room temperature. Then, the primary antibodie anti-LC3 (CST, Cat# 12741S, 1:200) were used to incubate the samples overnight at 4 °C in a humid chamber, and donkey anti rabbit IgG ALEXA flour 488 (1:1000, #a21206, Invitrogen) secondary antibody were used to incubate the samples for 1 h at room temperature. After three more washes, the stained nuclei were stained with Hoechst 33342 for 20 min. Images were taken using a confocal microscope (Nikon A1RSi, Tokyo, Japan) and analyzed using Software ImageJ (National Institutes of Health).

### 4.10. Western Blotting

Cells were lysed in a RIPA lysis (Beyotime Biotechnology, Shanghai, China) buffer containing mixed protease inhibitors on ice for 20 min. The cell lysates were collected by using cell scraper on ice and were centrifuged at 12,000 rpm for 15 min at 4 °C. The protein concentrations were detected by using the enhanced BCA Protein Assay kit (Beyotime Biotechnology, Shanghai, China), and the extracted protein solution was denaturalized with 5 × loading buffer at 100 °C. Total proteins (20–30 µg) were separated by electrophoresis on 8–12% SDS-PAGE gel and transferred to polyvinylidene fluoride (PVDF) membranes (Roche, Mannheim, Germany). Then, the membranes were blocked with 5% skim milk at room temperature for 2 h followed by overnight incubation with the primary antibodies at 4 °C. After recycling the primary antibody, the membranes were washed four times in TBST for 10 min each time. IgG (H + L) secondary antibody or HRP secondary antibodies were used to incubate the membranes for 1 h at room temperature. Finally, the protein bands were exposure by an ECL method. The relative proteins levels were analyzed by Image J software. Primary antibodies included anti-PPARβ/δ (Proteintech, Cat# 10156-2-AP, 1:1000); anti-GRP78 (abcam, Cat# ab21685, 1:1000); anti-Chop (CST, Cat# 2895S, 1:1000); anti-phospho-IRE-1α (S724) (abcam, Cat# ab48187, 1:1000); anti-IRE-1α (abcam, Cat# ab37073, 1:1000); anti-phospho-PERK (Thr98) (CST, Cat# 3179S, 1:1000); anti-PERK (CST, Cat# 3192S, 1:1000); anti-ATF6 (Proteintech, Cat# 24169-1-AP, 1:1000); anti-phospho-JUK (Thr183/Tyr185) (CST, Cat# 4668S, 1:1000); anti-JUK (CST, Cat# 9252S, 1:1000); anti-Caspase3 (CST, Cat# 9665S, 1:800); anti-pro Caspase12 (abcam, Cat# ab8117, 1:1000); anti-Caspase12 (abcam, Cat# ab62484, 1:1000); anti-LC3 I/II (Proteintech, Cat# 14600-1-AP, 1:1000); anti-Atg5 (Proteintech, Cat# 10181-2-AP, 1:1000); anti-Beclin1 (Proteintech, Cat# 11306-1-AP, 1:1000); anti-P62 (Proteintech, Cat# 18420-1-AP, 1:1000); anti-β Tubulin (Proteintech, Cat# 10094-1-AP, 1:5000); and anti- COXIV (CST, Cat# 11967S, 1:2000).

### 4.11. Reverse Transcription and Real-Time Quantitative PCR

Total RNA was isolated from treated-microglial cells by using Trizol reagent (Invitrogen Life Technologies, Carlsbad, CA, USA). The quality and quantity of total RNA were measured by Nano Drop 2000 spectrophotometer (Nano Drop Technologies, Thermo Scientific, Waltham, MA, USA). According to the manufacturer’s instructions, 1 µg total RNA was reverse-transcribed and synthesized the complementary DNA by using Prime Script TM RT Master Mix (TaKaRa, Tokyo, Japan). Real-time qPCR was carried out using SYBR^®^ Premix Ex Taq I (TaKaRa, Japan) following the instructions of reagent kit on a Quant Studio 5 Real-Time PCR System (Applied Biosystems, Waltham, MA, USA). The primer sequences of target genes were shown in Table 1. β-actin was regarded as the internal standard to normalize other target genes, and the 2(−ΔΔct) method was used to analyze the relative expression of the target genes. Each sample was run in triplicate, and each experiment was repeated at least thrice.

### 4.12. Transmission Electron Microscope Analysis

To observe the ultrastructural of endoplasmic reticulum and mitochondria in astrocytes, the ultrastructural analysis was performed as described previously [52]. All 1 mm^3^ hippocampus cubes were post fixed with ice-cold glutaraldehyde (2.5% in 0.1 M cacodylate buffer, pH 7.4) for 24 h, and then post fixed by immersion in OsO4 for 2 h. Next, the tissues were dehydrated in gradient acetone (from 50% to 100%) and embedded in epoxy resin. 1% osmium tetroxide and dehydrated through a graded series of ethanol. The area of stratum radiatum of CA1 of the hippocampus was selected to investigate the ultrastructural changes of endoplasmic reticulum and mitochondria. The ultrathin sections (70–90 nm thick) were cut by the ultramicrotome, placed on a copper grid, and stained with uranyl acetate and lead citrate. The stained sections were examined under a transmission electron microscope (JEM 1210; JEOL, Tokyo, Japan).

### 4.13. Transfection of Primary Astrocytes with siRNA

Astrocytes were seeded at the density of 10^4^ cells per well in six-well plates and transfected with siRNAs (50 nM, 200 nM, and 500 nM) targeting UCP2 using Lipofectamine Messenger MAX mRNA Transfection Reagent (Invitrogen, New York, NY, USA) according to the manufacturer’s instructions (sequences are listed in Table 2). More than 50% knockdown of the targeted proteins was observed after 500 nM siRNA treatment. Scrambled siRNA and target gene-specific siRNAs were purchased from Gene Pharma (Shanghai, China).

### 4.14. Flow Cytometry

Astrocytes were collected and washed twice with PBS and blocked by 0.1% Triton X-100 and 3% BSA in PBS. Astrocytes were then stained with AV-PI (BD Biosciences, La Jolla, CA, USA). Astrocytes were examined with a BD FACSVerse flow cytometer (BD Biosciences), and all of the tests were controlled by the homologous isotype control antibodies.

### 4.15. Intracellular ROS Assessment

The ROS levels were determined according to the manufacturer’s instructions of ROS assay kit (Jiancheng Bioengineering Institute, Nanjing, China). In cells, DCHF-DA is enzymatically hydrolyzed by intracellular esterases to form non fluorescent DCFH, which can’t penetrate the cell membrane and is then rapidly oxidized to form highly fluorescent DCF in the presence of ROS. The DCF fluorescence intensity emission was recorded at 530 nm (with 502 nm excitation) 30 min after the addition of DCHF-DA. After the corresponding treatment, astrocyte was collected and washed with serum-free DMEM. DCFH-DA probe were added to astrocytes, incubated at 37 °C for 30 min, and the ROS levels of astrocytes were determine by laser scanning confocal microscope (CarlZeiss LSM710, Zeiss, Germany) and Cytation5 cell imaging microplate detection system (BioTek, Winooski, VT, USA).

### 4.16. Glutathione Assay

The glutathione (GSH) content in the sample was detected according to the procedure described by the manufacturer of GSH and GSSG Assay Kit (Beyotime Biotechnology, Shanghai, China). Briefly, cell samples or GSH standard were loaded into the 96-well plates. The assay buffer (volume 150 μL) containing 5,5′dithiobis-2-nitrobenzoic acid, Ellman’s reagent (DTNB) was added to each of the wells and incubated in the dark on an orbital shaker for 5 min at 25 °C. Subsequently, 50 μL 0.5 mg/mL NADPH was added to each of the wells and the absorbance was measured at 405–414 nm. The difference in absorbance was calculated and GSH concentrations were determined based on a standard curve. The levels of GSH were reported as nmol/mg protein.

### 4.17. Determination of Mitochondrial Membrane Potential and ATP Content

Cellular MMP was measured using the mitochondrial membrane potential assay kit with JC-1 according to manufacturer’s instruction (Beyotime Biotechnology, Shanghai, China). JC-1 (10 μM) was used to incubate cells with the medium containing at 37 °C for 30 min and then, washed three times with 1× buffer solution in Primary astrocytes. ATP levels in cultured cells were detected by the ATP Assay Kit following the manufacturer’s protocol (Beyotime Biotechnology, Shanghai, China).

### 4.18. Chromatin Immunoprecipitation (ChIP) Assay

ChIP assays were performed to determine PPARβ/δ binding to UCP2 promoter region. Myc-PPARβ/δ was overexpressed in astrocytes by electrotransfection, then were treated with Cort and GW0742 as described previously. Cells in all groups were cross-linked in 37% formaldehyde for 10 min at room temperature, and the cross-linking reavtion was quenched by adding a final concentration of 0.125 M of glycine for 5 min. Cells were extracted in SDS lysis buffer containing Protease Inhibitor Mixture II. Chromatin was sheared by sonicating each sample fifty times for 20 s on ice. After centrifugation, the chromatin concentration and chromatin fragments were checked using Nanodrop and 2% agarose gel. The anti-Myc or anti-Flag antibody was added per sample and incubated overnight at 4 °C. Normal rat anti-IgG antibody was used as a nonspecific control. Protein G-agarose was added, and the sample was mixed for 4 h at 4 °C by rotation. Precipitated complexes were eluted in elution buffer and cross-linking was reversed by 5 M NaCl per sample. Coimmunoprecipitated DNA was purified according to the manufacturer’s directions. The relative abundance of PPARβ/δ precipitated chromatin binding site in the UCP2 promoter region was detected by qPCR. Site of UCP2 promoter located between −488 and −496 from the transcription starting site (Figure 4h), and the following primer set for promoter quantification: Forward: TCTTTTCTCTTCCCCGGAGT, Reverse: GCCAGAGGCAAAAAG.

### 4.19. Luciferase Reporter Assay

To determine the effect of PPARβ/δ agonist on UCP2 promoter transcriptional activity, Astrocytes were co-transfected with UCP2 promoter-luciferase reporter construct and Luciferase Reporter vector without UCP2 promoter. 24 h after cell transfection, we used corticosterone and/or GW0742 to treat astrocytes as described previously. In addition, the dual-luciferase reporter assay system (Promega, Madison, WI, USA) to measure luciferase activity. The firefly luciferase value validated the candidate target gene translation activity of UCP2.

### 4.20. Statistical Analysis

All experiments were independently repeated in triplicate at least, and all measurement data were presented as the mean ± SEM. Normality was tested using the Shapiro–Wilk test and homogeneity of variances was tested using the Levene test. Statistical analysis was measured with by one-way ANOVA with post-hoc Tukey’s multiple comparisons test and two-way ANOVA with post-hoc Bonferroni’s multiple comparisons test. *p*-values of less than 0.05 were considered statistically significant. GraphPad Prism 7.01 software was applied to analyze statistical significance. Electronic laboratory notebook was not used.

## 5. Conclusions

Our study identified the effect of PPARβ/δ on astrocytic damage in model conditions and the underlying molecular mechanism. We found that PPARβ/δ promoted the enhancement of mitophagy in astrocytes by targeting UCP2, and also demonstrated that PPARβ/δ-mediated mitophagy significantly inhibits the apoptosis of astrocytes, which induced by oxidative stress and ER stress. The present study provides a new insight for depression therapy.

## Figures and Tables

**Table 1:** Primers’ sequence.

Target Gene	Orientation	Primer Sequence (5’-3’)
UCP2	Forward	GCCACTTCACTTCTGCCTTC
	Reverse	GAAGGCATGAACCCCTTGTA
β-actin	Forward	AATCGTGCGTGACATCAAG
	Reverse	ATGCCACAGGATTCCATACC

**Table 2:** Primer’ sequence.

Target Gene	Orientation	Primer Sequence (5’-3’)
Si-UCP2	Forward	GCCACTTCACTTCTGCCTTC
	Reverse	GAAGGCATGAACCCCTTGTA

## Data Availability

The data that support the findings of this study are available from the corresponding author upon reasonable request.
